# Long-term physical functioning and quality of life after pelvic ring injuries

**DOI:** 10.1007/s00402-019-03170-2

**Published:** 2019-04-11

**Authors:** H. Banierink, I. H. F. Reininga, E. Heineman, K. W. Wendt, K. ten Duis, F. F. A. IJpma

**Affiliations:** 1 Department of Trauma Surgery, University Medical Center Groningen, University of Groningen, Groningen, The Netherlands; 2 Emergency Care Network Northern Netherlands (AZNN), Northern Netherlands Trauma Registry, Groningen, The Netherlands

**Keywords:** Pelvic ring injury, Functional outcome, Quality of life, Physical functioning, SMFA, EQ-5D

## Abstract

**Background:**

Pelvic ring injuries are serious injuries, often associated with substantial morbidity and mortality rates. The long-term consequences of these injuries might affect the patients’ personal life. Our aim was to assess the long-term effects of pelvic ring injuries on physical functioning and quality of life (QoL) using validated patient-reported outcome measures (PROMs) and comparing these results to normative data from the general population.

**Patients and methods:**

A retrospective cohort study was conducted on adults treated for pelvic ring injuries between 2007 and 2016. Demographics, fracture type, injury mechanism, treatment and complications were recorded. PROMs questionnaires concerning physical functioning (SMFA) and quality of life (EQ-5D) were used. Patients were divided according to their age (18–30, 31–64, 65 and older) and fracture type (Tile/AO type A, B or C). Differences in SMFA and EQ-5D scores of the operatively and non-operatively treated patients and between the study population and general population were analyzed.

**Results:**

A total of 413 patients were identified of which 279 were eligible for follow-up. One-hundred and ninety-two (69%) patients responded with a mean follow-up of 4.4 years. Patients reported a median score of 13.9 on the SMFA function index, 16.7 on the bother index, 12.5 on the lower extremity, 18.8 on the activities of daily living and 23.4 on the emotion subscale. A median EQ-5D score of 0.8 was reported. There was no difference in physical functioning and QoL between operatively and non-operatively treated patients. Comparison of these results to normative data of the general population revealed a significant (*P* < 0.05) decrease in physical functioning and QoL in patients with all types of pelvic ring injuries.

**Conclusion:**

Long-term physical functioning and QoL in patients who had sustained a pelvic ring injury seems fair, although significantly decreased in comparison with their peers from the general population.

## Background

Pelvic ring injuries have a prevalence of 20–37/100,000 in the general population and are often caused by severe accidents [1]. Most pelvic ring injuries are caused by (blunt force) high-energy trauma [2], with the majority of the causes being motor vehicle collisions [3, 4]. However, pelvic ring injuries in the elderly are often caused by low-energy accidents, such as a fall on a slippery surface.

Life-threatening situations can occur due to traumatic disruption of the pelvic ring [5]. The reported overall mortality varies from 5% in isolated pelvic ring injuries, up to 46% in poly-trauma patients [6, 7]. Patients who get through the initial hospital course following these injuries often have to endure a long period of impaired mobilization and intense rehabilitation.

Pelvic injuries do not only have a major impact in the short-term, but also long-term permanent limitations which can affect daily functioning. The latter includes gait impairment, chronic pelvic and back pain as well as delayed consequences of lumbosacral plexus injury [8], all of which may influence the patient’s quality of life [9].

Pelvic ring injuries occur in patients of all ages, with different comorbidities and physical conditions. The seminal work entitled ‘Fractures of the pelvis and acetabulum’ written by Tile et al. (page 361), clearly states that “adequate follow-up studies on pelvic ring fractures are lacking” [10]. This was our incentive to perform a large cohort study about the long-term personal and societal impact of these injuries using validated questionnaires (Short Musculoskeletal Function Assessment and EuroQol 5D).

Hence, the aim of this study was to provide an overview of the physical functioning and quality of life (QoL) of patients with pelvic ring injuries attending a level 1 trauma center over a period of 9 years. Additionally, the level of physical functioning and QoL of these patients were compared to normative data from the general Dutch population.

## Patients and methods

### Patients

All the adult patients (≥ 18 years of age) who had been treated for a pelvic ring injury at the Department of Trauma Surgery of the University Medical Center Groningen (UMCG) between January 2007 and January 2016 were approached for this study. The UMCG is a Level 1 trauma center and a secondary referral center for the treatment of pelvic injuries in the northern part of the Netherlands. Data about the patient’s characteristics were collected by reviewing each patient’s medical and operation records. Additional data were retrieved from the Dutch Trauma Registry, concerning injury severity in terms of the Abbreviated Injury Scale (AIS) [11] and Injury Severity Score (ISS) [12]. Subsequently, two trauma surgeons with ample experience in pelvic fracture surgery reassessed the radiographic images (plain anteroposterior, inlet and outlet radiographs and computerized tomography scans) of all the patients and classified the pelvic ring injuries into type A, B and C injuries (“Appendix”) according to the AO/OTA trauma pelvis and acetabulum manual [13]. Patients were divided according to their age (18–30, 31–64, 65 and older) and fracture type (Tile type A, B or C). The local Medical Ethical Review Board reviewed the methods employed and waived further need for approval (METc 2016.385).

### Long-term physical functioning and quality of life

Patients who had no cognitive disorders and were still alive in the follow-up period received a series of questionnaires by mail to assess long-term physical functioning and quality of life.

Physical functioning was measured with the Dutch version of the Short Musculoskeletal Function Assessment (SMFA-NL). The SMFA questionnaire consists of 46 items and was designed to assess the functional status of patients with various musculoskeletal disorders and injuries. The SMFA includes two indices: “function index” and “bother index” [14]. The Dutch version of the SMFA (SMFA-NL) has an additional four subscales that cover the physical functioning of all extremities, problems with daily activities and psychological aspects of functioning [15]. The scores vary from 0 to 100, with a higher score indicating a worse function. The SMFA scores of this study were compared to the normative data of the SMFA-NL in the general Dutch population [16].

Quality of life was assessed with the EuroQol 5D (EQ-5D). The EQ-5D is a brief questionnaire that measures health-related quality of life based on five dimensions of health: mobility, self-care, usual activities, pain/discomfort and anxiety/depression [17]. Patients can use the dimensions to delineate whether they have (1) no problems, (2) slight, (3) moderate, (4) severe or (5) extreme problems. The EQ-5D scores of this study’s population were compared to the normative data from the EQ-5D of the general Dutch population [18]. Moreover, physical functioning and quality of life between operatively and non-operatively treated patients were compared.

### Statistical analysis

Descriptive statistics were performed to present demographics, injury mechanism, fracture patterns and treatment methods. Means and standard deviations were calculated from the normally distributed data and the median and range from not-normally distributed data. To attain the SMFA-NL and EQ-5D data, the patients were divided according to their type of injury: type A, type B and type C. To analyze the association between fracture type and outcome with regard to physical functioning and quality of life, univariate analyses of variance (ANOVA) were performed. To compare the SMFA-NL and EQ-5D scores between operatively and non-operatively treated patients, Mann–Whitney *U* tests were performed. Additionally, the SMFA-NL and EQ-5D scores were compared to the age-matched normative data of the Dutch population using a manual T-test with pooled means and pooled SD’s. The data were analyzed using the IBM SPSS software, version 23.0 for Windows (IBM Corporation, Armonk, NY, USA). Statistical significance was accepted at *P* ≤ 0.05.

## Results

### Patients

**Fig. 1:**
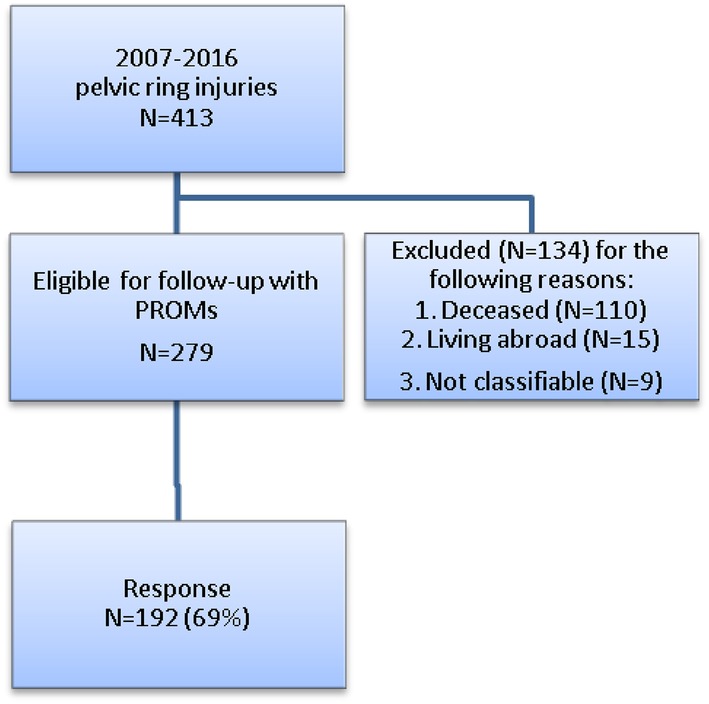
Flow-chart of patient inclusion for assessment of long-term physical functioning and quality of life after pelvic ring injuries

A total of 413 adults (≥ 18 years of age) with pelvic ring injuries were identified over a study period of 9 years (January 2007 until January 2016) of which 279 (68%) patients were eligible for follow-up by means of patient-reported outcomes. The main reason for exclusion was that 110 (26%) of the patients had died at long-term follow-up. A total of 192 patients (69%) at a mean follow-up of 4.4 ± 2.6 years after the pelvic ring injury responded. The other 84 patients (31%) declined to participate or did not respond (Fig. 1). A non-response analysis was performed which showed no significant differences between the responders and non-responders, except for a difference in age (57 vs. 47). Table 1 demonstrates the demographic and injury characteristics of the 192 responders, divided into the different fracture types (A, B and C).

**Table 1:** Individual and injury characteristics of the responders

	Type A (*n* = 75)	Type B (*n* = 99)	Type C (*n* = 18)	All patients (*n* = 192)
Follow-up in years				
Mean ± std.	4 ± 2.6	4.4 ± 2.6	5.2 ± 2.8	4.3 ± 2.7
Age (years) at injury				
Median (range)	60 (20, 93)	51 (18, 89)	38 (19, 62)	54 (18, 93)
Male, *n* (%)	32 (43)	60 (61)	16 (89)	108 (56)
Injury mechanism				
LET	38 (51)	22 (22)	0 (0)	60 (31)
HET	37 (49)	77 (78)	18 (100)	132 (69)
Treatment				
Conservative	71 (95)	74 (75)	5 (28)	150 (78)
Operative	4 (5)	25 (25)	13 (72)	42 (22)
ISS				
Median (range)	9 (4, 43)	13 (4, 75)	21 (11, 43)	13 (4, 75)
ISS ≥ 16, *n* (%)	29 (39)	44 (44)	14 (78)	87 (45)
Highest pelvis AIS				
Median (range)	2 (2, 5)	3 (2, 5)	3 (2, 4)	2 (2, 5)
AIS 2, *n* (%)	58 (78)	43 (43)	4 (22)	105 (55)
AIS 3, *n* (%)	16 (21)	47 (48)	13 (72)	76 (39)
AIS 4, *n* (%)	0 (0)	4 (4)	1 (6)	5 (3)
AIS 5, *n* (%)	1 (1)	5 (5)	0 (0)	6 (3)

*LET* low-energy trauma, *HET* high-energy trauma, *ISS* injury severity score, *AIS* abbreviated injury score

### Physical functioning and quality of life

The results of the patient-reported outcomes are presented in Table 2. Overall, patients with pelvic injuries, regardless of the type of injury, gave fair scores for all the SMFA parts (Table 2). They reported moderate limitations with, respectively, a median of 13.9 on the function index, 12.5 on the lower extremity and 18.8 on the activities of daily living (ADL) subscale. Patients with type A pelvic injuries reported slightly higher scores on most SMFA indices and subscales in comparison with type B and C injuries. However, no significant differences were found in the function and bother indices and lower extremity, ADL and emotion subscales of the SMFA between patients with type A, B and C injuries. The only significant difference was between type A and type C injuries regarding the SMFA upper extremity subscale (*P* = 0.047). The three SMFA questions with the highest scores (mean of more than 2.5 on a scale from 1 to 5), in relation to decreased physical functioning, were regarding feeling disabled, feeling tired and the effect of doing too much in one day which could affect what the patient is able to do the next day. Concerning the lower extremity subscale of the SMFA, the three questions with the highest scores (mean of more than 2 on a scale from 1 to 5) concerned difficulties with bending and kneeling down, moving after sitting or lying and walking with a limp.

**Table 2:** SMFA-NL and EQ-5D outcomes

	Type A	Type B	Type C	All patients
SMFA				
Function Index (*n* = 165)				
Median (range)	15.4 (0, 92)	13.6 (0, 83)	9.0 (0, 51)	13.9 (0, 92)
Mean ± std.	25.2 ± 27.4	20.7 ± 20.7	17.5 ± 17.2	21.9 ± 22.9
Bother Index (*n* = 192)				
Median (range)	19.8 (0, 88)	14.6 (0, 81)	16.7 (0, 65)	16.7 (0, 88)
Mean ± std.	28.8 ± 27.2	24.6 ± 23.3	22.9 ± 21.6	26.1 ± 24.7
Lower extremity (*n* = 171)				
Median (range)	13.5 (0, 96)	10.4 (0, 94)	11.5 (0, 58)	12.5 (0, 96)
Mean ± std.	24.3 ± 27.9	18.8 ± 22.1	17.2 ± 18.4	20.5 ± 23.9
Upper extremity (*n* = 192)				
Median (range)	0 (0, 96)	0 (0, 79)	0 (0, 33)	0 (0, 96)
Mean ± std.	16.4 ± 26.9	9.6 ± 19.6	2.3 ± 7.9	11.5 ± 22.4
ADL (*n* = 178)				
Median (range)	21.3 (0, 96)	18.6 (0, 90)	13.1 (0, 65)	18.8 (0, 96)
Mean ± std.	30.8 ± 31.5	26.3 ± 25.1	22.2 ± 22.5	27.5 ± 27.4
Emotion (*n* = 192)				
Median (range)	25 (0, 84)	21.9 (0, 84)	18.8 (0, 68.6)	23.4 (0, 84)
Mean ± std.	29.5 ± 22.3	25.9 ± 20.6	27.1 ± 21.5	27.4 ± 21.3
EQ-5D (*n* = 191)				
Median (range)	0.807 (− 0.109, 1)	0.805 (− 0.134, 1)	0.843 (0.298, 1)	0.805 (− 0.134, 1)
Mean ± std.	0.742 ± 0.275	0.764 ± 0.264	0.792 ± 0.214	0.758 ± 0.264

*ADL* activities of daily living

Overall, all patients who had sustained a pelvic ring injury, irrespective of the type, reported a reasonable QoL (Table 2) with a mean EQ-5D score around 0.8 (on a scale from − 0.329 to 1, with a higher score indicating a better QoL). Furthermore, there were no significant differences in EQ-5D scores between the various types of pelvic ring injuries.

Also, no differences in physical functioning and QoL were found between the conservatively and operatively treated patients.

### SMFA and EQ-5D scores compared to normative data from the Dutch population

SMFA and EQ5D scores were compared to normative data from the general Dutch population. Regarding the SMFA, middle- and older-aged patients who had sustained either a type A, type B or type C pelvic ring injury, reported significantly more physical impairment on the function index in comparison to their peers in the general population (Table 3). The results of the SMFA bother index were similar for patients with type A and B injuries. Patients who had sustained a pelvic injury and were aged > 30 reported significantly worse physical functioning on most subscales compared to the normative data of the general Dutch population (Table 4). These results apply to all the types of pelvic ring injuries, especially regarding the lower extremity and daily activity subscale. All the patients in this study cohort generally reported worse mean function (22 vs. 12), bother (26 vs. 13), lower extremity (21 vs. 11), daily activity (28 vs. 12) and emotional (27 vs. 21) outcome scores on the SMFA after 4 years of follow-up in comparison to the normative data.

**Table 3:** SMFA index scores compared to normative data of the Dutch population

Patients (*n*)	Fracture type^a^	Dutch population^a^	*P* value	Difference on a scale from 0 to 100 (%)^b^
Function				
Age	*Type A*			
18–30 (*n* = 8)	16.1 ± 20.1	10.1 ± 12.4	> 0.05	6.0
31–64 (*n* = 28)	20.7 ± 21.3	12.2 ± 13.4	< **0.05**	8.5
≥ 65 (*n* = 19)	35.6 ± 35.2	12.9 ± 13.5	< **0.05**	22.7
	*Type B*			
18–30 (n = 14)	17.0 ± 21.0	10.1 ± 12.4	< **0.05**	6.9
31–64 (*n* = 56)	20.4 ± 19.6	12.2 ± 13.4	< **0.05**	8.2
≥ 65 (*n* = 24)	23.7 ± 23.3	12.9 ± 13.5	< **0.05**	10.8
	*Type C*			
18–30 (*n* = 4)	11.0 ± 11.8	10.1 ± 12.4	> 0.05	0.9
31–64 (*n* = 11)	21.4 ± 18.5	12.2 ± 13.4	< **0.05**	9.2
Bother				
Age	*Type A*			
18–30 (*n* = 9)	17.6 ± 21.6	9.0 ± 14.5	> 0.05	8.6
31–64 (*n* = 30)	25.1 ± 24.7	15.1 ± 18.6	< **0.05**	10.0
≥ 65 (*n* = 37)	34.5 ± 29.4	15.3 ± 18.7	< **0.05**	19.2
	*Type B*			
18–30 (*n* = 15)	21.0 ± 25.7	9.0 ± 14.5	< **0.05**	12.0
31–64 (*n* = 59)	23.8 ± 22.6	15.1 ± 18.6	< **0.05**	8.7
≥ 65 (*n* = 27)	28.3 ± 23.8	15.3 ± 18.7	< **0.05**	13.0
	*Type C*			
18–30 (*n* = 5)	23.8 ± 22.0	9.0 ± 14.5	< **0.05**	14.8
31–64 (*n* = 12)	24.5 ± 22.3	15.1 ± 18.6	> 0.05	9.4

^a^Mean SMFA scores and standard deviation

^b^Decrease in physical functioning compared to normative data of the Dutch population

**Table 4:** SMFA subscale scores compared to the Dutch population normative data

Patients (*n*)	Fracture type^a^	Dutch population^a^	*P* value	Difference on a scale from 0 to 100 (%)^b^
Lower extremity				
Age	*Type A*			
18–30 (*n* = 8)	11.7 ± 17.7	7.6 ± 12.9	> 0.05	4.1
31–64 (*n* = 29)	18.8 ± 21.3	10.8 ± 14.4	< **0.05**	8.0
≥ 65 (*n* = 21)	36.7 ± 35.0	13.6 ± 14.8	< **0.05**	23.1
	*Type B*			
18–30 (*n* = 15)	15.7 ± 21.6	7.6 ± 12.9	< **0.05**	8.1
31–64 (*n* = 56)	17.5 ± 20.4	10.8 ± 14.4	< **0.05**	6.7
≥ 65 (*n* = 24)	23.8 ± 26.1	13.6 ± 14.8	< **0.05**	10.2
	*Type C*			
18–30 (*n* = 5)	13.8 ± 16.2	7.6 ± 12.9	> 0.05	6.2
31–64 (*n* = 12)	20.1 ± 19.6	10.8 ± 14.4	< **0.05**	9.3
Upper extremity				
Age	*Type A*			
18–30 (*n* = 8)	9.4 ± 16.5	5.5 ± 10.0	> 0.05	3.9
31–64 (*n* = 56)	10.1 ± 16.3	5.7 ± 11.4	< **0.05**	4.4
≥ 65 (*n* = 37)	23.0 ± 33.5	7.2 ± 13.8	< **0.05**	15.8
	*Type B*			
18–30 (*n* = 15)	1.9 ± 5.6	5.5 ± 10.0	> 0.05	3.6^▲^
31–64 (*n* = 59)	9.7 ± 20.8	5.7 ± 11.4	< **0.05**	4.0
≥ 65 (*n* = 27)	13.6 ± 21.1	7.2 ± 13.8	< **0.05**	6.4
	*Type C*			
18–30 (*n* = 5)	0 ± 0	5.5 ± 10.0	> 0.05	5.5^▲^
31–64 (*n* = 12)	3.5 ± 9.7	5.7 ± 11.4	> 0.05	2.2^▲^
Daily activities				
Age	*Type A*			
18–30 (*n* = 8)	18.6 ± 26.9	9.0 ± 14.9	> 0.05	9.6
31–64 (*n* = 28)	25.4 ± 27.1	14.0 ± 18.1	< **0.05**	11.4
≥ 65 (*n* = 27)	40.0 ± 35.3	14.0 ± 17.3	< **0.05**	26.0
	*Type B*			
18–30 (*n* = 14)	21.3 ± 28.4	9.0 ± 14.9	< **0.05**	12.3
31–64 (*n* = 28)	25.5 ± 23.1	14.0 ± 18.1	< **0.05**	11.5
≥ 65 (*n* = 26)	30.8 ± 27.9	14.0 ± 17.3	< **0.05**	16.8
	*Type C*			
18–30 (*n* = 4)	15.0 ± 18.0	9.0 ± 14.9	> 0.05	6.0
31–64 (*n* = 11)	26.9 ± 23.8	14.0 ± 18.1	< **0.05**	12.9
Emotion				
Age	*Type A*			
18–30 (*n* = 9)	24.3 ± 16.7	21.0 ± 16.6	> 0.05	3.3
31–64 (*n* = 30)	25.3 ± 20.9	22.0 ± 17.7	> 0.05	3.3
≥ 65 (*n* = 37)	34.1 ± 24.0	19.8 ± 17.1	< **0.05**	14.3
	*Type B*			
18–30 (*n* = 15)	24.2 ± 25.5	21.0 ± 16.6	> 0.05	3.2
31–64 (*n* = 59)	26.2 ± 20.8	22.0 ± 17.7	> 0.05	4.2
≥ 65 (*n* = 26)	26.4 ± 17.9	19.8 ± 17.1	> 0.05	6.6
	*Type C*			
18–30 (*n* = 5)	26.9 ± 20.0	21.0 ± 16.6	> 0.05	5.9
31–64 (*n* = 12)	29.2 ± 22.7	22.0 ± 17.7	> 0.05	7.2

^a^Mean SMFA scores and standard deviation

^b^Decrease in physical functioning compared to normative data of the Dutch population, except for scores indicated with ▲, which indicates the score is higher compared to that of the Dutch population

With respect to the EQ-5D, significant differences were found between type A and type B fractures in the 31–64 and ≥ 65 age groups compared to their peers in the general Dutch population, whereby the patients who had sustained a severe pelvic ring injury reported a relatively lower quality of life (Table 5).

**Table 5:** EQ-5D scores compared to the Dutch population normative data

Patients (*n*)	Fracture type^a^	Dutch population^a^	*P* value	Difference on a scale from − 0.329 to 1 (%)^b^
EQ-5D				
Age	*Type A*			
18–30 (*n* = 9)	0.817 ± 0.133	0.894 ± 0.154	> 0.05	5.8
31–64 (*n* = 30)	0.767 ± 0.241	0.853 ± 0.178	< **0.05**	6.5
≥ 65 (*n* = 38)	0.712 ± 0.317	0.865 ± 0.170	< **0.05**	11.5
	*Type B*			
18–30 (*n* = 15)	0.781 ± 0.285	0.894 ± 0.154	< **0.05**	8.5
31–64 (*n* = 59)	0.779 ± 0.255	0.853 ± 0.178	< **0.05**	5.6
≥ 65 (*n* = 29)	0.733 ± 0.284	0.865 ± 0.170	< **0.05**	9.9
	*Type C*			
18–30 (*n* = 5)	0.766 ± 0.291	0.894 ± 0.154	> 0.05	9.6
31–64 (*n* = 12)	0.803 ± 0.189	0.853 ± 0.178	> 0.05	3.8

^a^Mean EQ-5D scores and standard deviation

^b^Decrease in quality of life compared to the Dutch population

## Discussion

The aim of this study was to provide an overview of the long-term physical functioning and quality of life (QoL) of patients following pelvic ring injuries. Additionally, their level of physical functioning and quality of life were compared to the normative data of the general Dutch population. The results of this study show fair long-term physical functioning and QoL after all types of pelvic ring injuries (Table 2). No clinically relevant differences in long-term physical functioning and quality of life were found between patients who had sustained A, B or C type pelvic ring injuries. However, comparisons with the normative data of the Dutch population showed a significant decrease in physical functioning and QoL in all types of pelvic ring injuries and in all age groups (Tables 3, 4, 5). Moreover, the fact that research has shown that injured patients initially report better pre-injury health status compared to the general population [19] even more suggests that the impact of pelvic ring injuries on physical functioning and quality of life may even be larger than the results of this study indicate. A few small cohort studies have reported the results of physical functioning after pelvic ring injuries. Lefaivre et al. found poorer physical functioning after type B and type C pelvic ring injuries on applying the SMFA [20], with a mean of 52.12 points for type B injuries and 62.57 points for type C injuries, compared to 20.7 and 17.5, respectively, in our study population. The SMFA bother index scores were 51.51 for type B and 64.18 for type C injuries compared to 24.6 and 22.9 in our study population. However, it is hard to compare these results because only 38 patients participated in their study, none of whom had type A injuries and all the patients were treated operatively. Our large cohort of both conservatively and operatively treated patients, on the other hand, reflects daily clinical practice.

In our study, the pelvic ring injury patients demonstrated substantially lower physical functioning (mean SMFA function score 22 vs. 12; bother 26 vs. 13; lower extremity 21 vs. 11; daily activity 28 vs. 12; emotion 27 vs. 21) and quality of life (mean EQ-5D 0.76 vs. 0.87) after 4 years of follow-up in comparison to their peers from the general population. The decrease in physical functioning at follow-up, as measured by the SMFA, mainly strikes patients aged > 30 years and especially patients aged ≥ 65 (Tables 3, 4). This could probably be explained by the fact that, even though more young people sustain the relatively severe type B and C injuries, they tend to have better recovery capacity and coping mechanisms compared to older patients. Older patients often sustain the more stable type A injuries, but are more likely to have pre-existing comorbidities. Together with the age-related vulnerability and limited rehabilitation capacity, this may explain the fact that elderly patients had significantly decreased physical functioning after a pelvic ring injury compared to the younger patients.

To the best of our knowledge, only a few papers compared validated PROMs regarding physical functioning and QoL following pelvic ring injuries with normative data [19, 20]. In one of these studies, by Hoffmann et al., patients with LC pelvic injuries reported worse daily activity (23.9 vs. 11.9), emotional (32.7 vs. 20.5), lower extremity (25.7 vs. 13.6), function (21.8 vs. 12.7) and bother (24.2 vs. 13.8) outcome scores on the SMFA after 2 years of follow-up in comparison to the normative data [21].

The patients in our study still had a decreased QoL (median EQ-5D of 0.8) due to their pelvic ring injuries several years after the accident. The decrease in QoL, as found in our study, seems to be in line with the previous literature. A study by Harvey-Kelly et al. showed a significant decrease in all five domains of the EQ-5D score (median 0.67) at 1 year follow-up after traumatic pelvic injury, compared to the pre-injury status [22]. Dienstknecht et al. divided their patients into three groups namely, isolated anterior pelvic ring injuries, isolated posterior pelvic ring injuries and combined anterior and posterior pelvic ring injuries. They found poorer quality of life after a minimum of 10 years of follow-up in patients with posterior pelvic ring injuries and combined anterior and posterior pelvic ring injuries, as measured by the SF-12 [23]. Moreover, Holstein et al. found that older patients had a higher likelihood of reduced quality of life following complex trauma and surgery [9].

There has been an increase in the use of generic outcome instruments. The overall validity, reliability and responsiveness of pelvic outcome instruments have not been established and the information in the existing literature is inadequate for surgeons or patients about the functional outcomes after these injuries [24]. The EQ-5D and SMFA-NL are valid and reliable questionnaires that provide a generalized (functional) personal outcome score. They were used here because of these characteristics and the ability to compare our data with the normative data from the Dutch population. Moreover, these questionnaires were considered to complement each other in specific aspects following pelvic injuries. Historically, outcome reports after pelvic injuries mainly focused on radiographic measures. However, the patients’ own perception with regard to social, physical and emotional challenges is of greater importance.

Some strong points and some limitations of this study should be addressed. The strengths of this study include the size of the patient cohort, the relatively long follow-up period and the high response rate (69%). Whereas other studies mostly used non-validated measures to evaluate outcomes after pelvic ring injuries, this is one of the few studies that used several validated questionnaires to assess long-term physical functioning and quality of life in a large cohort of patients who had sustained a pelvic ring injury. The use of validated questionnaires enabled comparison of the results with normative data from the general population. Most studies which evaluated functional outcomes after pelvic ring injuries excluded pelvic type A injuries caused by low-energy traumas; these type of injuries were included in our study because they form the largest part of the entire population with pelvic ring injuries. A possible limitation is the fact that the study suffers from heterogeneity in terms of fracture patterns and the presence of associated injuries, although this is a clinical reality in patients suffering from pelvic ring injuries. Second, the retrospective cross-sectional study design has inherent restrictions. Despite this, we believe that several critical important issues were addressed.

In conclusion, it seems just to address long-term patient-reported physical functioning and quality of life of patients who have sustained a pelvic ring injury, especially as it can be substantially lower in comparison with their age-matched peers from the general population. This indicates that pelvic ring injuries have a significant personal as well as societal impact, even years after the injury occurred. Further prospective research with validated PROMs is necessary to assess the course of physical functioning and quality of life at regular time intervals, from the pre-injury status to a number of years post-injury.

## Appendix

See Table 6.

**Table 6:** Fracture classification with subtypes according to the AO/OTA method

	All patients (%) (*n* = 413)
61—A	184 (45)
61 A1.1	2
61 A1.2	–
61 A1.3	1
61 A2.1	20
61 A2.2	136
61 A2.3	16
61 A3.1	2
61 A3.2	3
61 A3.3	4
61—B	174 (42)
61 B1.1	10
61 B1.2	11
61 B2.1	104
61 B2.2	9
61 B2.3	7
61 B3.1	5
61 B3.2	5
61 B3.3	23
61—C	46 (11)
61 C1.1	6
61 C1.2	4
61 C1.3	16
61 C2.1	3
61 C2.2	7
61 C2.3	4
61 C3.1	1
61 C3.2	3
61 C3.3	1
No classification^a^, *n* (%)	9 (2)

^a^Could not be classified due to lack of computer tomography imaging
